# Cognitive impairment and the associated risk factors among the elderly in the Shanghai urban area: a pilot study from China

**DOI:** 10.1186/2047-9158-1-22

**Published:** 2012-11-26

**Authors:** Jun-Peng Zhuang, Gang Wang, Qi Cheng, Li-Ling Wang, Rong Fang, Li-Hua Liu, Ran Xiong, Yu Zhang, Ya-Xing Gui, Wen-Yan Kang, Hui-Dong Tang, Sheng-Di Chen

**Affiliations:** 1 Department of Neurology & Institute of Neurology, Ruijin Hospital affiliated to Shanghai Jiao Tong University School of Medicine, Shanghai, 200025, China

**Keywords:** Cognitive impairment, Prevalence, Risk factors

## Abstract

**Objectives:**

Our study aimed to investigate the prevalence of cognitive impairment(CI) and the associated risk factors among elderly people in Shanghai urban area, China.

**Methods:**

A population-based survey was conducted among people aged 55 years or older in urban areas of Shanghai. Face-to-face interviews were carried out to collect information including demographic characteristics, medical history, and medication use, etc. The validated Chinese version of the Mini-Mental State Examination(MMSE) was used to screen subjects with CI, and the criteria of CI were adjusted for education levels.

**Results:**

A total of 3,176 home-living residents (≥55 years old) were included in the study. Among them, 266 people (102 men and 164 women) were identified as cognition impaired, with a prevalence of 8.38% (266/3,176, 95% CI: (8.26, 8.49)) for both genders, 9.21% (102/1,107,95% CI: (9.18, 9.33)) for men and 7.93% (164/2,069, 95% CI: (7.80, 8.09)) for women, respectively. Furthermore, we found that several significant risk factors, including social factors(education, number of children, marriage status, and family structure), physiological factors (age, blood glucose level, and obesity), factors on living styles(physical exercise, diet & chronic diseases), and genetic factor(ApoE), associated with CI onset.

**Conclusions:**

This study confirms the high prevalence of CI among the elderly population in the Shanghai urban in China, similar to previous epidemiologic studies in Western countries. The putative risk factors associated with CI merit further investigated.

## Introduction

Numerous studies have shown that the prevalences of Alzheimer’s disease(AD) in China is comparable with the data in European and North American [1,2]. Meanwhile, Pre-dementia sydromes comprised of different types of cognitive impairments (CI) are very worthwhile to assess the prevalence and incidence in populations. But only limited studies are conducted to examine the epidemiology of CI in China with the largest aging population so far. Given that the population in China is aging rapidly, and that the prevalence of CI increases with age, it has been expected that the number of prevalent cases of CI would increase dramatically in the present, especially in largest cities, for example, Shanghai. However, most available investigations are with small sample size or hospital-based [3,4], both of which make it difficult to generalize the results to whole China. On the other hand, limited studies on CI has significantly impeded the preventions of AD and recognition of the impacts of pre-dementia syndrome under China’s current Socio-Economic-Status context [5,6].

Therefore, on the basis of our previous study for CI and associated factors among the elderly in the Shanghai suburb [7], we estimated the prevalence of CI as well as associated factors for CI among home-living population (≥55 years old) in urban areas of Shanghai, China.

## Methods

### Study locale and subjects

A population-based survey was conducted in three randomly selected communities in Luwan District of Shanghai City in 2009. In this study, a total of 3,176 residents aged 55 years or older were face-to-face interviewed and screened by trained neurologistand raters.

### Cognition impairment diagnosis

A validated Chinese version Mini-Mental State Examination (MMSE) [2] was given to every participant by neurologists or trained raters for evaluations. The diagnosis criteria for CI employed was primarily based on current medical conditions (mainly according to patients’ complaining, recent laboratory tests and neuroimaging if available), past & family diseases history (especially dementia, hypertension, diabetes mellitus, hyperlipidemia, etc.), and mental status evaluation at the time of survey in the form of MMSE score with participants’ education taken into account (Diagnosed as CI if MMSE ≤ 17 for illiterates; MMSE ≤ 20 for primary school graduates(≥6 years of education); MMSE ≤ 24 for junior school graduates or above(≥9 years of education)) [8,9]. However, as this was a screening study rather than rigorous diagnosis, the researchers didn’t apply the neuroimaging tests to all the participants.

### Covariates collection

Beside MMSE screening, face-to-face interviews were conducted to collect subjects’ demographic information, life style, living environment, diet habit, past & family diseases history (especially CI, hypertension, diabetes mellitus, hyperlipidemia, current diseases, medications, etc.), psychological status (depression, anxiety, insomnia, neurosis, etc.).

One free brief physical examination was conducted to measure subjects’ weight, height, blood pressure, abdominal circumference and blood glucose levels. Furthermore, a sample of peripheral blood was collected from each voluntary individual upon their approval for ApoE gene assessment. Genotyping analysis of ApoE [10] was performed as previously described.

All data were gathered and screened for validity by senior neurologists. Totally, 306 records were randomly selected for double-check by in-person visiting or telephone-checking. The study was approved by the Research Ethics Committee, Ruijin Hosptial, Medicine School of Shanghai Jiaotong University, China.

### Statistical analysis

All data analyses were performed using SPSS. Chi square test and analysis of variance (ANOVA) were used to test between-group differences in categorical and continuous variables, respectively.

To explore the potential associated factors for CI, we fit thirteen simple linear regression models using MMSE score as dependent variable while social, physicological and genetic actors as dependent variables if the between-group differences are statistically significant. We used R^2^ value to rank and identify the associated factors for CI.

## Results

### Demographic characteristics

Among 3,176 subjects, 1,107 were males and 2,069 females (average age for males: 70.54 ± 9.25; average age for females: 69.26 ± 9.65). Within all the respondents, 2,517 were married and lived with their spouses at the time of study while the other 659 were either widowed, divorced, never married or refuse to tell their marriage status. Within all the subjects, 1,019 claimed themselves having education less than 6 years; 1,005 admitted to have more than 10 years of education; and 1,152 sitting in between.

### Prevalence of CI

Among all the 3,176 participants with valid MMSE scores, 266 (8.38%, 95CI:(8.26,8.49)) were diagnosed as cognitive impaired according to the pre-set diagnosing criteria. The prevalence was 9.21% (95 CI: (9.18, 9.33) for males while 7.93% (95 CI:(7.80, 8.09)) for females. However, there was no significant cognitive impairment rate difference on gender (Pearson Chi-square *P* value 0.201). Furthermore, the prevalence remains at 3.49% in the 55 ~ 70 age group compared with those older than 70 with a prevalence of more than 10% (Table 1).

**Table 1:** Demographic distribution of subjects by gender in general samples and CI samples

	**Male**	**Female**	**Total**
	**(*n *= 1,107)**	**(*n *= 2,069)**	**(*n *= 3,176)**
Number of people	1,107	2,069	3,176
Age (years, mean ± SD)	70.54 ± 9.25	69.26 ± 9.65	69.70 ± 9.53
Eduacation (years) Pearson’s P value <0.001			
<6	228	791	1,019
6-10	408	744	1,152
>10	471	534	1,005
CI (n)	102	164	266
Percentage (%)	9.21%	7.93%	8.38%
Age (years, mean ± SD)	73.31 ± 11.03	72.84 ± 12.87	73.02 ± 12.21
Eduacation (years) Pearson’s P value <0.001			
<6	18	98	116
6-10	59	45	104
>10	25	21	46

### Associated factors associated with CI

Negative associated factors included Live alone (*Pearson’s *P* value = 0.004; Fisher’s *P* value = 0.007), more number of children(≥3) (*Pearson’s *P* value < 0.001; Fisher’s *P* value < 0.001), Aging (* Pearson’s *P* value <0.001), Diabete (OR = 1.42, Pearson’s *P* value = 0.007; Fisher’s *P* value = 0.009), Obesity (BMI ≥30) (OR = 1.63, Pearson’s *P* value=0.048; Fisher’s *P* value =0.06), Dysuria (OR =1.59 for chronic dysuria), Constipation (OR=1.40 for constipation, Pearson’s *P* value = 0.006; Fisher’s *P* value = 0.008), and ApoE homozygous (OR=3.00, Pearson’s *P* value = 0.007). Positive associated factors composed of stable marriage (* Pearson’s *P* value < 0.001; Fisher’s *P* value < 0.001), High educations (with education period more than 12 years) (*Pearson’s *P* value <0.001), Frequent physical exercise (OR=2.34, Pearson’s *P* value < 0.001), Drinking coffee (OR=2.48, Pearson’s *P* value = 0.030; Fisher’s *P* value=0.030), Sleeping(continuous sleeping time ≥4 hours) (Pearson’s *P* value = 0.029; Fisher’s *P* value = 0.043).

### The influence of associated factors on the MMSE

Finally, 13 separate single variable linear regression models were constructed with MMSE score as the dependent variable. The 13 risk factors were then the independent variables respectively, with only “diabetes” replaced by “blood glucose”. By comparing the R^2^ or adjusted R^2^ value, all the 13 associated factors were ranked (Table 2). The R^2^ represented the independent contribution to the variance of dependent variable and MMSE score. The sequence of the risk factors were (from the most influential to the least): education, age, number of children, marriage status, blood glucose, physical exercise, family structure, dysuria, coffee drinking, constipation, continuous sleeping time, ApoE allele, and obesity. However, these thirteen risk factors only accounted part (30%) of CI onset. Many more potential aspects, though not significantly standing out in this research (hypertension, smoke, etc.) might also conduce to the onset of CI (Table 3).

**Table 2:** Simple variable linear regression output

**Dependent variable**	**MMSE Score**				
**Independent variables**	**Regression coefficient**	***P *value**	**R**^**2**^	**Adjusted R**^**2**^	**Ranking of influence to CI**
Education	1.455	<0.001	0.217	0.216	**1**
Age	−0.18	<0.001	0.194	0.193	**2**
Number of Children	−1.143	<0.001	0.170	0.169	**3**
Marriage Status	−2.317	<0.001	0.058	0.058	**4**
Blood Glucose	−0.169	<0.001	0.009	0.009	**5**
Physical Exercise	0.593	<0.001	0.009	0.009	**5**
Family Structure	−1.171	<0.001	0.008	0.008	**7**
Dysuria	−1.094	<0.001	0.007	0.006	**8**
Coffee Drinking	1.254	<0.001	0.005	0.004	**9**
Constipation	−0.778	<0.001	0.004	0.004	**10**
Sleeping time (≤ 4 hours)	−1.678	0.001	0.003	0.003	**11**
ApoE Allele	−0.376	0.035	0.002	0.002	**12**
Obesity	−0.634	0.041	0.002	0.001	**13**

**Table 3:** Multiple regression model output

**Dependent variable**	**MMSE Score**		
**Independent variables**	***P *value**	**R**^**2**^	**Adjusted R**^**2**^
Cumulative of all the 13 Significant Variables (including Education/Age/Number of Children/Marriage Status/Blood Glucose/Physical Exercise/Family Structure/Dysuria/Coffee Drinking/Constipation/Sleeping time/ApoE Allele/Obesity)	<0.001	0.304	0.297

## Discussion

This study is the first to reveal the prevalence of CI and the associated factors among elderly people in Shanghai urban over the past decade. The prevalence of CI(2-3%) is significant lower compared to the similar investigations from Eureapean countries and Northern American according to the Chinese representive studies before 2001 years [1,2]. However, recent studies showed that the truths might probably be the opposite with a more relative high value. The present study proposed that in urban of China, the prevalence of CI were closer to that of most Western countries [11,12] and our previous study in suburban of China [7]. For this disparity, except for ethnic difference (Chinese Han ethnicity and Caucasian), the most important possible explanation might that the changes of the proportion of aging peoples result in the increasing ratio of CI: the senile population increased markedly (over 7% in total populations) compared to the proportions of the total in China since 2001.

Furthermore, the thirteen associated factors screened out might have profound implications to the individuals, the governments and even the whole Society. Among all the factors locked, education, age, number of children, or ApoE genotype are those nearly impossible so far for human beings to alter or reverse. “Age” itself is actually a universal risk factor to nearly every disease except for those only attack the young. However, family structure, marriage status and a healthy life style with enough exercise, frequent coffee drinking along with active interventions against chronic diseases might help to prevent the onset or slow down the progress of CI [13-15]. According to the risk factor ranking, the prevalence of CI might be reduced by encouraging elder people living with their spouse or offsprings [16]. Living with relatives meant more communications and more interactions both physically and mentally [17]. Drinking more coffee, having enough sleep and consulting the physicians once the problems like prostate hypertrophy (which is the main cause of dysuria) or constipation appear are also beneficial for CI preventing. Maintaining an appropriate blood glucose level might also be preventive by resisting the degeneration of the vascular system, sensitizing the utilization of the glucose and thus satisfying the energy demand of the local neural systems [18]. Exercise stimulates the execration of 5-HT which sustains the functional activity of neurons and maintains a high spirit [15]. Additionally, the results revealed that the ApoE allele is a significant risk factor for CI similar with previous studies [19-21]. However, in considering its weighing on the influence of CI onset it is far less enough to be recommended as a diagnosis predictor clinically. As a clinical physician facing the patients, sociological and physiological factors are, if not more, as important as molecular genetics.

However, with the regression approach we totally screened out thirteen significant objective factors associated with CI among the sample population. Combined together, these thirteen factors could only explain 30% of the CI occurrence. One explanation is that we might have lost other important risk factors like hypertension and smoking. In fact, according to our results, though there is an augmentation trend of CI rate in those who smoke or have hypertension, they are not statistically significant either by Chi-square test or in a regression model. Thus, we presumed that CI is influenced by multiple factors. Different populations have varied sets of significant risk factors. The results we obtained only represent the urban population in Luwan, Shanghai.

The present study is limited by its cross-sectional nature and only included one urban areas. Determining of CI mainly depended on MMSE scores rather than neuroimage results and other neuropsychiatric scales. Additionally, the methods for investigate the prevalence of CI with these two different cut-off criteria, including the attained education level(AEL) and MMSE cut-off score of 23/24 or 24/25 without education adjustment CI [8]. Therefore, we need modify the methods for study and reasonability compare with Western countries which most employed the latter methods. Additionally, we found that the relative highly educated sample in the present study rather than our previous relatively low educated have a relative high prevalence of CI, except for sample discrepancy, it seem like that high education is not an independent protective factor which will be affected by other factors. Therefore, further prospective study for the Chinese people requires to be investigated at more large scale in the future.

## Abbreviations

CI: Cognitive Impairment; MMSE: Mini-Mental State Examination; AD: Alzheimer’s disease; AEL: Attained Education Level.

## Competing interests

The authors declare that they have no competing interests.

## Authors' contributions

J-P Zhuang made contributions to conception and design, acquisition of data(statistical analysis), and in drafting the manuscript. G Wang made contributions to conception and design, acquisition of data and in drafting the manuscript. Q Cheng, L-L Wang, R Fang, L-H Liu, R Xiong, Y Zhang, Y-X Gui and W-Y Kang participated in the design and execution. S-D Chen and H-D Tang were the general supervision of the research group, acquisition of funding, and involved in revising it critically for important intellectual content. All authors read and approved the final manuscript.
